# Detection of somatic epigenetic variation in Norway spruce via targeted bisulfite sequencing

**DOI:** 10.1002/ece3.4374

**Published:** 2018-09-05

**Authors:** Katrin Heer, Kristian K. Ullrich, Manuel Hiss, Sascha Liepelt, Ralf Schulze Brüning, Jiabin Zhou, Lars Opgenoorth, Stefan A. Rensing

**Affiliations:** ^1^ Conservation Biology Faculty of Biology Philipps University Marburg Marburg Germany; ^2^ Department of Ecology Faculty of Biology Philipps University Marburg Marburg Germany; ^3^ Plant Cell Biology Faculty of Biology Philipps University Marburg Marburg Germany; ^4^ College of Life Sciences Shaanxi Normal University Xi'an China; ^5^ Department of Evolutionary Genetics Max Planck Institute for Evolutionary Biology Ploen Germany; ^6^ State Key Laboratory of Grassland Agro‐Ecosystems School of Life Sciences Lanzhou University Lanzhou China; ^7^ BIOSS Biological Signaling Studies University of Freiburg Freiburg Germany

**Keywords:** acclimation, epigenetics, exome capture, gene body methylation, *Picea abies*

## Abstract

Epigenetic mechanisms represent a possible mechanism for achieving a rapid response of long‐lived trees to changing environmental conditions. However, our knowledge on plant epigenetics is largely limited to a few model species. With increasing availability of genomic resources for many tree species, it is now possible to adopt approaches from model species that permit to obtain single‐base pair resolution data on methylation at a reasonable cost. Here, we used targeted bisulfite sequencing (TBS) to study methylation patterns in the conifer species Norway spruce (*Picea abies*). To circumvent the challenge of disentangling epigenetic and genetic differences, we focused on four clone pairs, where clone members were growing in different climatic conditions for 24 years. We targeted >26.000 genes using TBS and determined the performance and reproducibility of this approach. We characterized gene body methylation and compared methylation patterns between environments. We found highly comparable capture efficiency and coverage across libraries. Methylation levels were relatively constant across gene bodies, with 21.3 ± 0.3%, 11.0 ± 0.4% and 1.3 ± 0.2% in the CG, CHG, and CHH context, respectively. The variance in methylation profiles did not reveal consistent changes between environments, yet we could identify 334 differentially methylated positions (DMPs) between environments. This supports that changes in methylation patterns are a possible pathway for a plant to respond to environmental change. After this successful application of TBS in Norway spruce, we are confident that this approach can contribute to broaden our knowledge of methylation patterns in natural tree populations.

## 1 INTRODUCTION

Forest trees are particularly challenged by anthropogenically induced climate change due to their sessile, long‐lived lifestyle and long generation times. Thus, the question whether and how trees are able to cope with these rapid climatic changes is of utmost importance (Aitken, Yeaman, Holliday, Wang, & Curtis‐McLane, 2008). Local adaptation of tree populations, as one possible coping strategy, is always a trade‐off between gene flow and the strength of selective pressure and might thus be slowed down if gene flow is extensive (Savolainen, Pyhäjärvi, & Knürr, 2007). Furthermore, in long‐lived organisms, long generation times can prevent fast adaptational processes. In recent years, evidence has accumulated that, independent of genetic changes, epigenetic mechanisms like DNA methylation and histone modifications are involved in the response of organisms to environmental changes (Richards et al., 2017). At least among nonmodel organisms, DNA methylation patterns were studied most intensively and were shown to be influenced by environmental cues in plants with diverse life history traits such as annual plants, clonally reproducing perennials or trees (Gugger, Fitz‐Gibbon, Pellegrini, & Sork, 2016; Keller, Lasky, & Yi, 2016; Platt, Gugger, Pellegrini, & Sork, 2015; Richards, Schrey, & Pigliucci, 2012; Verhoeven, Jansen, van Dijk, & Biere, 2010). When translating into phenotypic changes, epigenetic mechanisms essentially contribute to phenotypic plasticity (Herrera & Bazaga, 2013; Kooke et al., 2015; Nicotra et al., 2010) and, consequently, represent a possible mechanism for achieving a rapid response to environmental change (Bossdorf, Richards, & Pigliucci, 2008). Epigenetic changes *via* priming (e.g., changes of DNA methylation that alter the transcriptional response) can occur during embryogenesis as well as at later life stages (Sani, Herzyk, Perrella, Colot, & Amtmann, 2013). Such priming might be particularly relevant for long‐lived plant species, independent of whether these changes are heritable (generative/adaptation) or act during the lifetime (somatic/acclimation) only. Thus, an in‐depth understanding of the role of epigenetic mechanisms in trees might crucially contribute to our understanding of the strategy of these long‐lived organisms for coping with environmental change and might help explain their ecological and evolutionary success.

Norway spruce (*Picea abies* (L.) Karst) is one of the few tree species for which epigenetic phenomena have already been investigated. Early studies focused solely on the phenotype, and found that the offspring of Norway spruce trees that originated from high altitudes, but were grown at lower altitudes, had a growth rhythm that was more similar to trees from lower altitudes (Skrøppa, 1994; Skrøppa & Kohmann, 1997). Later studies shifted their attention to the underlying molecular mechanisms and investigated gene expression and small RNAs. Climatic conditions during embryogenesis had a strong influence on the expression of candidate genes, including genes involved in small RNA processing pathways (Yakovlev, Asante, Gunnar, Junttila, & Johnsen, 2011; Yakovlev, Fossdal, & Johnsen, 2010; Yakovlev et al., 2014). In combination, the phenotypic response to environmental conditions and the linked responses at the sRNA and gene expression level indicate that epigenetic mechanisms might be involved in acclimation in Norway spruce, and a logical next step was thus a detailed look at the effects of environmental change on methylation patterns. However, due to the lack of genomic resources and the limited possibilities to set up experiments with trees, there are only few studies on single‐base resolved methylation patterns in trees so far (Bräutigam et al., 2017; Gugger et al., 2016; Platt et al., 2015). Consequently, our current knowledge on changes of methylation patterns in response to climatic differences comes mainly from a small number of model plant species, most prominently the annual weed *Arabidopsis thaliana*. Therefore, despite accumulating high‐resolution data on methylation patterns and other epigenetic mechanisms, the ecological and evolutionary consequences of epigenetics remain unresolved mainly for two reasons. First, it is unclear in how far findings in *A. thaliana* might be transferrable to other plants in general and to trees in particular. For example, in comparison with other plant species, *A. thaliana* has an extremely small genome with an exceptionally low content of DNA methylation and transposable elements (Alonso, Pérez, Bazaga, & Herrera, 2015). Furthermore, due to its annual life cycle, it has very short generation times. In addition, most studies on *A. thaliana* and a number of crop species took place under controlled conditions and experimental setups, which might not necessarily reflect processes under natural conditions and longer time spans. Secondly, most epigenetic studies in nonmodel species with clear ecological questions and in natural settings are based on global methylation patterns or methylation‐sensitive amplified polymorphism (MSAPs; e.g., Raj et al., 2011; Sáez‐Laguna et al., 2014), which are far less informative than whole‐genome bisulfite sequencing (WGBS) approaches. Some recent studies on methylation patterns in ecological model species used reduced representation sequencing approaches based on restriction enzymes (Bräutigam et al., 2013; Gugger et al., 2016; van Gurp et al., 2016; Platt et al., 2015; Trucchi, Mazzarella, Gilfillan, Schoenswetter, & Paun, 2016). However, these methods do not allow targeting of specific genomic regions of interest. In this study, we sought to establish a cost‐efficient method to screen methylation patterns in Norway spruce at single‐base pair resolution by targeted bisulfite sequencing (TBS).

In recent years, genomic resources have become available for a growing number of species, among them forest trees like Norway spruce (Nystedt et al., 2013). However, sequencing costs for full genome or methylome population studies are still prohibitive for extremely large and complex genomes such as those of conifer species that, for example, contain a high proportion of transposable and repetitive elements. Due to their size and complexity, none of the conifer genomes are assembled at the chromosome level, consist of an extremely large number of scaffolds, and are often not well annotated which further complicates WGBS studies. In such cases, TBS appears to be the most promising method that allows a reduction in the analyzed genome space. Here, we focus on gene body methylation (GBM) mainly for two reasons: First, GBM might be of particular interest for the response of plants to the environment (Bewick et al., 2017; Jiang et al., 2014; Platt et al., 2015). Second, downstream analysis depends on annotated sequences which are most reliable for a set of high confidence genes in Norway spruce. However, limiting the analysis of methylation patterns to GBM implies that also informative and likely relevant methylation patterns up‐ and downstream of the coding space will not be captured. Targeting the expressed regions of the genome has already been applied successfully in other conifer species (Neves, Davis, Barbazuk, & Kirst, 2013; Pavy et al., 2016). Lately, several approaches where developed that apply exome capture to bisulfite converted DNA (Bainbridge et al., 2010; Lee et al., 2011). Here, we use the SeqCap Epi kit (NimbleGen) exome capture bead array which has mostly been used for methylation studies in humans, but has also been successfully applied in other organisms such as rat and maize (Li, Song, et al., 2015; Li, Suzuki, et al., 2015; Masser et al., 2016).

While TBS now provides the opportunity to tackle methylation at single‐base pair resolution, we are still confronted with the challenge of disentangling genetic and epigenetic effects when studying methylation patterns under natural environmental conditions. A large proportion of methylation patterns specifically in gene bodies is genetically controlled in plants (Dubin et al., 2015), and environmental conditions might influence both genetic variation via selection and epigenetic variation via acclimation. To circumvent this problem, a number of studies have therefore focused on populations that either reproduce asexually or have naturally low levels of genotypic diversity (Richards et al., 2012; Sáez‐Laguna et al., 2014; Verhoeven et al., 2010), which permits to assess directly the impact of environmental conditions on methylation. However, most tree species are outcrossing and characterized by high levels of genetic diversity. To overcome these problems, we took advantage of a Norway spruce seed orchard established in 1990. For this seed orchard, cuttings from autochthonous source trees from the Bavarian Forest National Park were planted in a productive environment in lowland Bavaria. As a result, we can now utilize the original trees (ortets) located at around 1,200 m above sea level (a.s.l.) and cuttings (ramets) grown at around 500 m a.s.l. to study changes in methylation patterns that occur during the lifetime of a tree in response to contrasting environmental conditions.

With this pilot study, we provide the first data on the feasibility of TBS in a conifer tree species. We critically discuss the advantages and disadvantages of this approach and thereby provide a backbone for future studies. Based on the obtained data, we provide insights into methylation patterns in gene bodies of conifers and compare methylation patterns of initially genetically identical trees that grew under different environmental conditions for 24 years. If environmental conditions have an impact on GBM, we would expect differentially methylated positions (DMPs) in relevant loci between ramets and ortets. Specifically, we would expect DMPs in genes that contribute to acclimation or adaptation to climatic conditions at different elevations, for example, genes related to phenology and cold stress.

## 2 METHODS

### 2.1 Study site and sampling

The dataset was based on four Norway spruce trees (tree IDs: 65, 67, 68, 72) from a natural stand situated at about 1,200 m a.s.l. in the Bavarian Forest National Park, Germany (49.094607°, 13.290166°, Supporting information Figures S1 and S2). In 1990, twigs had been collected and grafted onto rootstocks in a seed orchard in Übersee, Germany, located at 520 m a.s.l (47.83361°, 12.4575°, Supporting information Figures S1 and S3). Rootstocks were obtained from four‐year‐old seedlings from a seed lot from Fichtelgebirge, Germany. The source trees from the natural stands will be referred to as ortets, the clonal trees in the seed orchard as ramets, and the respective pairs as genets.

The two locations have a very distinct climate. The site in the Bavarian Forest had a mean annual temperature of 4.7°C and a mean annual precipitation of 1,474 mm for the years 2000–2012 (Bässler, 2004). The climatic station in Chieming (same elevation as Übersee, in a distance of about 15 km) recorded a much higher mean temperature of 8.7°C and 1,110 mm of mean annual rainfall for the same time period (Supporting information Figure S4).

Needles of the ortets and ramets were sampled at the same day and time at both locations in April 2014 under very similar weather conditions. All trees were sampled before bud burst took place. Sampling during the same phenological phase might be important as methylation status was shown to change between bud burst and bud set in *Castanea sativa* (Santamaría et al., 2009). Using needles from the previous vegetation period, such effects should be avoided. Needles were immediately dried and stored on silica gel until DNA extraction. The genetic identity of the ortets and their ramets was confirmed with five variable microsatellite markers (Rungis et al., 2004). In addition, we genotyped ortets and ramets with ~2,000 SNPs (Heer et al., 2016) and found them to be identical for all positions. These SNPs were also used to determine genetic relatedness among the sampled individuals (Supporting information Figure S5).

### 2.2 Probe design

The probe design was based on a set of 26,437 genes (86.9 Mb) that were previously predicted with high confidence (HC genes, see Nystedt et al., 2013). This way, we did not cover the entire coding space of the Norway spruce genome, but reduced the risk of targeting pseudogenes. Probes were designed by NimbleGen using the genomic scaffolds as reference. Only probes that mapped uniquely to the targeted region in the genome were included in the final design. The SeqCap Epi kit follows a first‐convert‐then‐capture approach. The probes target both strands of bisulfite‐treated genomic DNA and capture all states of unmethylated to fully methylated DNA. As methylation in the CG context is typically much more frequent than in the CHG and CHH context, the design only considers C‐T wobble positions in the CG context. So far, no bias has been detected with this method (Li, Suzuki, et al., 2015; Masser et al., 2016).

### 2.3 Capture protocol

We followed the protocol of Roche NimbleGen SeqCap Epi with a few modifications outlined below. DNA was extracted with peqGOLD Plant DNA Mini Kit following the instructions of the manufacturer. DNA concentrations were determined using Hoechst 33258 nucleic acid stain in a FLUOstar Omega microplate reader (BMG Labtech). To obtain an average DNA fragment size of 180–220 bp, we sheared 1 μg of DNA and 5.8 μl of the bisulfite conversion control in a total volume of 130 μl 1 ×  TE by sonication in a Covaris S220 Focused‐ultrasonicator (Covaris, Woburn, MA, USA) in a Covaris microTUBE. Samples were subjected to sonication for a total of 200 s, alternating between 60‐s sonication intervals and 60‐s pause intervals, and a last sonication interval of 80 s. After two sonication intervals, the microTUBES were centrifuged for about 10 s. For the construction of the sequencing libraries, we followed the manufacturer's instructions of the KAPA library preparation kit, including the end repair of the 3′ and 5′ overhangs, an A‐tailing reaction, and ligation of adapters. Afterward, the libraries were bisulfite‐converted with the EZ DNA Methylation Lightning kit (Zymo Research), amplified with 12 PCR cycles, and purified using the Agencourt AMPure XP Kit (Beckman Coulter). Prior to hybridization, we added the Bisulfite Capture Enhancer and the universal and index oligos to the bisulfite‐converted libraries and dried them in a speed vacuum concentrator in tubes with open lids. For the hybridization, the libraries were kept in a thermocycler at 47°C for 72 hr. Afterwards, captured DNA was washed and recovered with capture beads, followed by a second amplification step of 16 PCR cycles. The quality of the libraries was controlled after fragmentation, after pre‐LM PCR and after post‐LM PCR on a Bioanalyzer using an Agilent High Sensitivity DNA chip.

After capture enrichment, four of the libraries (Tree ID 65 and 67) were sequenced with an Illumina HiSeq 2500 instrument resulting in ~25 million single‐end 100‐bp reads. About 35 million single‐end 150‐bp reads were added to that by sequencing on a HiSeq 3000. The remaining libraries were completely sequenced on the HiSeq 3000 to obtain ca. 60 million reads each (ENA http://www.ebi.ac.uk/ena/run accessions ERR2591764:ERR2591771).

### 2.4 Sequence analysis and capture performance

Reads were quality filtered with Trimmomatic 0.33 (Bolger, Lohse, & Usadel, 2014). We removed remaining adapters, bases at the leading and trailing end with a quality below 10, and bases with an average quality below 20 in a sliding window of 5 bp. Finally, we removed reads of less than 36 bases length.

Reads were mapped with bismark v0.16.1 (Krueger & Andrews, 2011) against an in silico bisulfite‐converted reference that consisted of the pre‐mRNAs of the 26,437 high confidence genes and 1,000 bp before and after the annotated mRNA (130 Mbp in total). The seed was set to 15 with one mismatch allowed, and we adjusted the functions that define the minimum score required for a valid alignment (–score_min L,‐1,‐1). Mapped BAM files are available under the ENA project PRJEB26494.

The conversion rate of the bisulfite reaction was determined as the proportion of Cs that were successfully converted to Ts in the unmethylated Lambda DNA that was added as conversion control in each sample. In order to determine the efficiency of the capture approach, we evaluated the coverage across the probe regions, and across the targeted genes for each library. We used the “genomeCoverageBed” tool from the bedtools software suite v2.26.0 (Quinlan and Hall 2010) with the option – bga to obtain this information from the bam files obtained *via* bismark. The resulting coverage files contained information on the site‐specific coverage across the 26,437 genes of the reference for each library. To obtain the coverage across those regions that were directly targeted by probes, the coverage files were intersected with a GFF file that delimited the probe regions as provided by NimbleGen with “intersectBed” from the bedtools software suite. We then determined which proportion of the genes or probe regions was covered with reads using coverage thresholds from 1 to 100.

We used bismark methylation extractor to obtain data on the overall percentage of methylated Cs per context as well as on the methylation percentage for each covered C using the weighted DNA methylation method (#C/(#C+#T), Li et al., 2014). The latter information was used for further analysis with the R package MethylKit (Akalin et al., 2012). First, we filtered the data with thresholds for a minimum and maximum coverage of 8 and 100, respectively, to select positions with reasonable coverage.

### 2.5 Analysis of methylation patterns

Metagene plots of DNA methylation across gene bodies (between the 5′‐ and 3′‐ends of the mRNA including introns, and thereby limited to the regions covered by probes) were produced by first splitting each gene body into 20 equally long bins with “makewindows” from the bedtools software suite. Subsequently, each bin per gene (ordered from 5′ to 3′) was intersected with the site‐specific coverage files and only those bins were retained that showed a mean coverage ≥8 and ≤100. The retained bins were further intersected with the site‐specific methylation percentage files per C context (CG, CHG, CHH) with “intersectBed” from the bedtools software suite and only those bins retained which at least contained one site‐specific methylation (note that also 0% methylation per site will be reported by the bismark methylation extractor if the coverage falls within the specified borders). Further, for each bin, the mean methylation percentage was calculated and the corresponding bins (ordered from 5′‐ to 3′‐prime) were piled up to represent methylation across gene bodies per C context.

Based on this subset, we conducted a principal component analysis (PCA) to analyze the variance of the samples’ methylation profiles for each context. Second, we used the function *get.methylDiff* to select differentially methylated positions between ortets and ramets and defined thresholds of >25% difference between ortets and ramets and a *q* value < 0.01. We considered only those positions in the final analysis where methylation differed by >10% in the same direction for each ortet/ramet pair. For this set of DMPs, we conducted a Gene Ontology (GO) bias analysis to test which GO terms were relatively more or less abundant compared to the GO terms for the entire set of HC genes. Annotation of all HC genes was obtained from Heer et al. (2016). The GO bias analyses were conducted following Widiez et al. (2014, see supplementary material therein) using the R package GOstats (Falcon & Gentleman, 2007).

## 3 RESULTS

### 3.1 Capture design, performance, and reproducibility

The probe design covered 73.2 Mbp (84%) of 86.9 Mbp of the HC genes with a total of 82,368 probe regions with an average length of 902.5 ± 983.7 bp. Single probes were isothermal and had a length of 50–105 bp. Of the 16% not covered (including 250 genes with no probes), 3.4 Mbp is due to N stretches (gaps) and 23 kbp due to repeats. The probe design is available from Roche (OID42175 or PICAB_UMR).

We obtained between 47.1 and 66.0 million reads per library (mean 61.6 ± 5.9 million) that were mapped with an average efficiency of 42.86 ± 3.08% in Bismark. The conversion rate ranged from 98.9% to 99.5% among libraries.

**Figure 1:**
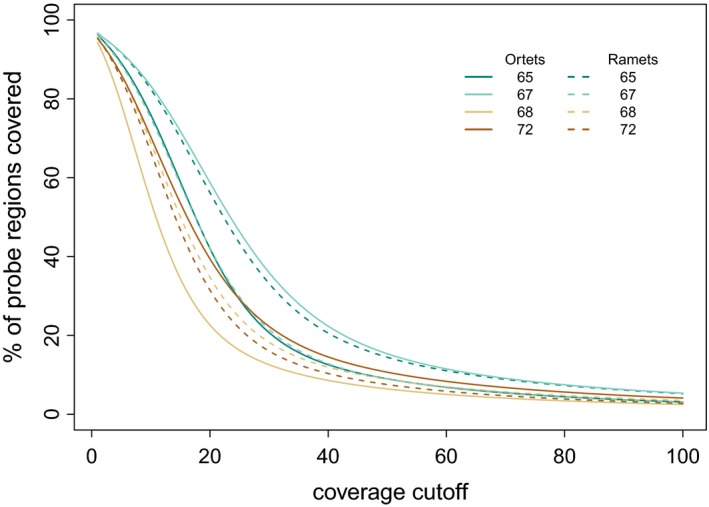
Capture efficiency of the targeted bisulfite sequencing approach in Norway spruce. The graph depicts the proportion of the regions targeted by capture probes that are covered with at least the read number indicated on the *x*‐axis. Colors represent clone IDs. Ortets are represented by straight lines, ramets by dashed lines

For each library, 97.6 ± 0.1% of the high confidence genes, and 98.3 ± 0.4% of the probes were covered with at least one read (Table Supporting information S1). A large proportion (72.0 ± 8.9%) of the regions directly targeted by probes were covered with >10 ×  reads, but this proportion quickly declined with higher coverage thresholds (Figure 1). When we extended this to the total length of the genes that were provided as reference for the probe design, 73.0 ± 8.2% of the 86.9 Mb were covered at least 10 ×  (Supporting information Figure S6). Comparing the libraries restricted to genes with at least a mean coverage of 10 ×  showed an overlap of 70.68% (18,686 genes).

### 3.2 Methylation patterns within genets and between environments

**Figure 2:**
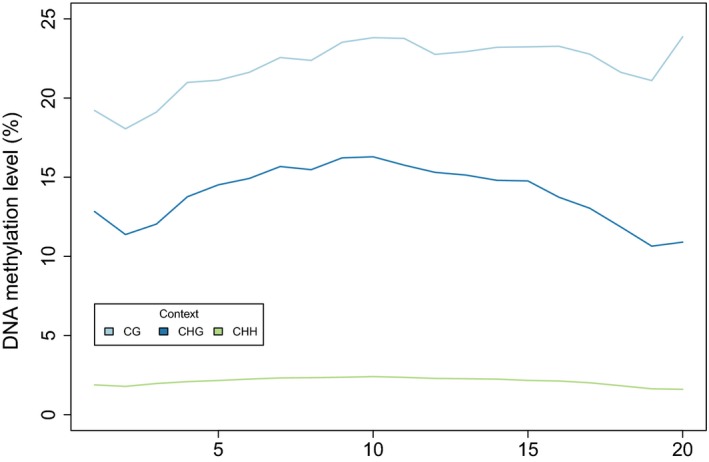
Distribution of DNA methylation within genes of Norway spruce for each context across genes. Each gene was divided into 20 bins of equal size across from the transcriptional start site to the transcriptional termination site covering exons and introns

**Figure 3:**
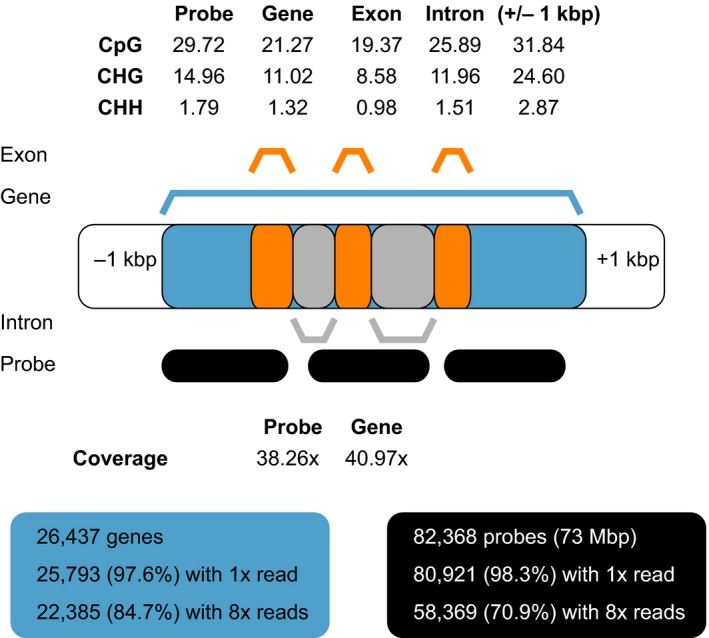
Visualization of an archetypical gene of Norway spruce with capture probes. The average gene is shown as blue bar with the average number of three exons (orange) and three probe regions (black) covering the genic region. All features are drawn to scale referenced by the 1 kbp upstream and downstream. Average context‐dependent percentage methylation of probes, genic regions, exons, and off‐target (1 kbp up‐/downstream) regions is shown above the gene. Average read coverage of genes and probes is shown below the gene. The boxes below indicate the proportion of genes and probe regions that are covered at different coverage depth

Methylation levels varied slightly across gene bodies, and were highest at the center and in the end in the CG context, highest in the center for CHG, and relatively constant for CHH (Figure 2). Methylation levels exhibited low levels of variation across all libraries, with a mean methylation level of 21.3 ± 0.3% (CG), 11.0 ± 0.4% (CHG), and 1.3 ± 0.2% (CHH) across the 20 bins that covered the full length of genes, and across all libraries. Methylation was lower when only coding regions were considered, and substantially higher in introns and UTRs 1 kbp upstream and downstream (Figure 3).

**Figure 4:**
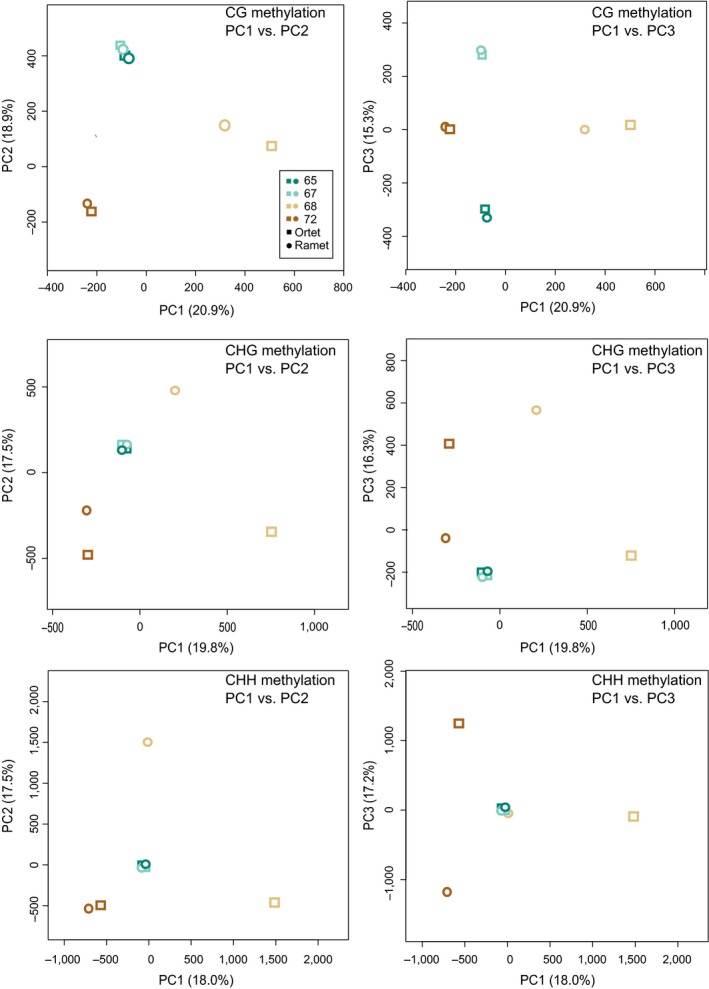
Principal component analysis (PCA) on methylation profiles of Norway spruce ortets and ramets for the CG, CHG, and CHH context, respectively. For each PCA, the left graph depicts the first vs. second PC, the right graph the first vs. third PC. Colors represent clone IDs. Ortets are represented by quadrats, ramets by triangles

While CG methylation seems to be similar between ortet and ramet based on the PCA, we found relatively large differences for two of the four ortet‐ramet pairs for methylation in the CHG and CHH context (Figure 4), specifically for individual 68 (all principal components (PC)s) and 72 (2nd and 3rd PCs). The first three PCs together explained 52.7%–55.1% of the variation.

For the analysis of DMPs, we considered 0.67, 1.12, and 4.85 million Cs in the CG, CHG, and CHH context, respectively. Most DMPs were found in the CHG context (225 DMPs), while only four could be identified in the CHH context (Table 1, Supporting information TableS2). The GO bias analysis of the DMPs across all three contexts showed an enrichment of biological process (BP) terms describing biotic interactions (such as cellular response to virus, modification by symbiont of host morphology or physiology), regulation of metabolism, and development (such as organ development, reproductive system development, Supporting information Figure S7).

**Table 1:** Total analyzed and differentially methylated positions in Norway spruce. The total analyzed positions include those Cs where all libraries had read coverage between 8 ×  and 100 ×. DMPs show significant differences in methylation between ortets from the Bavarian Forest National Park and ramets from the seed orchard in Übersee using MethylKit. Out of those, a subset showed a difference of >10% for each ortet‐ramet comparison

Methylation context	CG	CHG	CHH
# of analyzed positions	674.241	1.189.147	4.846.316
# of DMPs (MethylKit)	324	558	6
# of DMPs > 10% difference (proportion of total Cs in this context in parentheses)	105 (0.016%)	225 (0.019%)	4 (0.000083%)

## 4 DISCUSSION

With this pilot study, we successfully applied the SeqCap Epi kit for the first time in a conifer species, which are characterized by exceptionally large and complex genomes. We thus show that TBS is a feasible approach to reduce genomic complexity and target‐specific genomic regions even with a rudimentary draft genome. Specifically, we obtained data for 97% of the high confidence genes available from the Norway spruce draft genome, out of which 18,686 (70%) were covered in all libraries with a minimum 10 ×  coverage. Below, we discuss our results in detail, highlight the benefits and limitations of this method when applied in conifer species, and outline how the experimental design of this pilot study can be optimized for future studies with larger sample size.

The recently published methylome of Norway spruce (Ausin et al., 2016) and two studies on GBM that included conifer species (Bewick et al., 2017; Takuno, Ran, & Gaut, 2016) allow us to compare our exome capture results. Global methylation across the entire genome was very high in Norway spruce due to the high number of TEs. GBM was determined at ~55% in the CG context, ~25%–30% in the CHG context, and 1.5% in the CHH context (Ausin et al., 2016). Similarly, Takuno et al. (2016) observed methylation percentages of 46%, 42%, and 2% in gene bodies of the respective contexts for *Pinus taeda*. Values from both studies are much higher than what we discovered. Still, in accordance with these results, we detected CG and CHG methylation at almost equal levels in gene bodies, and a much lower percentage of CHH methylation. Whether the differences in the absolute CG and CHG methylation level have to be attributed to the different methods for library preparation and sequencing, different subset of genes, or the different individuals, will have to be determined in future studies.

Although exome capture already greatly reduced the sequencing effort, bisulfite sequencing requires a relatively high number of reads due to reduced mapping efficiency and for an exact determination of the methylation percentage. In our study, we were able to map about 43% of the reads which is at the lower end but still within the expected range for bisulfite sequencing studies (WGBS in Norway spruce: 56%, Ausin et al., 2016; SeqCapEpi in maize: 24%–57%, Li, Suzuki, et al., 2015). To correctly determine the percentage of methylation at a given locus, studies usually work with thresholds of 8–15 × . This still means that methylation levels cannot be estimated with high accuracy, specifically if methylation levels are very low or high. As we ultimately aim at investigating methylation patterns in natural populations and thus, in much higher number of individuals, the current cost for TBS can still be limiting. A sensible approach would be to first identify DMRs or DMPs in a smaller number of individuals or in pools of individuals with a large exome capture design as used in this study (or, in species with smaller genomes, with WGBS). In a second step, targeting these regions with a smaller subset of probes would be a compromise that permits to interrogate regions of interest in a high number of individuals with acceptable sequencing costs. Such approaches should not solely focus on GBM, but ideally cover DMRs across the entire genome. Next to GBM, it can be expected that DMRs in regulatory regions are particularly informative. Costs per sample could be further reduced by pooling several individually labeled libraries in the same capture reactions to use the relatively expensive probes pools more efficiently.

As expected, we found varying degrees of environmental influence on methylation patterns depending on the level of data resolution. At the global level, the methylation percentage between the clones from the two environments was largely identical. Thus, on this level methylation patterns remained largely stable over a period of 24 years despite strong climatic differences (Supporting information Figure S4). To our knowledge, there are now other studies that investigated methylation changes at single‐base pair resolution over such a time frame. In contrast to our results, other studies in diverse plant species have shown that global methylation levels tend to be higher in more stressful environments. For example, methylation was higher in alpine vs. subalpine habitats of *Betula ermanii* (Wu, Yi, et al., 2013) or in accessions from colder vs. warmer environments in *A. thaliana* (Dubin et al., 2015). Thus, we assume that environmental conditions were not particularly stressful in neither of the sites during the time since the ramets were planted.

On the level of the PCA analysis, no clear environmental pattern was evident either, although in two of four ramet‐ortet comparisons pronounced differences in the CHG and CHH context were found. While this is indicative for more stable methylation in the CG context vs. a higher number of idiosyncratic changes in the CHG and CHH context, it does not warrant an interpretation regarding the environmental influence. Given that single PCs did not explain more than 20% of the variation and that we analyzed only 8 libraries, this clearly indicates that potentially much variation is found between individuals as well as between groups of individuals.

As we had only a low number of genets in our study, we decided to approach the analysis of DMPs conservatively and only selected those where methylation changed in the same direction between low and high elevations for further analyses. This way, we could determine in total 334 DMPs between environments. For example, DMPs in the CG context were enriched for metabolic and developmental terms, among them flower and xylem development, phloem and xylem histogenesis, and response to toxic substances. The few CHH DMPs were exclusively enriched for stomatal complex morphogenesis (Supporting information Figure  S3). Many of these functions are linked to traits and processes that are relevant for plants under stressful conditions, such as the regulation of stomatal conductivity and xylem vulnerability (Brodribb, McAdam, Jordan, & Martins, 2014). This suggests that changes in the methylation status of specific gene bodies changed in response to differing climatic conditions, and might thus contribute to acclimation in trees. Based on tree ring data, we know that growth is drastically reduced at the high elevation plot compared to low elevations in the same area (data not shown). Given that conditions are even more favorable for growth at the seed orchard, we assume that such a drastic change of environmental cues might contribute to changes in methylation patterns.

Besides differences in environmental conditions, a number of other factors could potentially influence the degree to which methylation patterns differ between ortets and ramets. For example, Burian, Barbier de Reuille, and Kuhlemeier (2016) investigated how somatic mutations are distributed and accumulate in *A. thaliana* and extrapolated their results to trees based on the distribution of axillary meristems. They predicted that somatic mutations are mostly limited to small sectors of trees and thereby increase the genetic diversity among branches and the seeds generated from them. However, first genomewide studies of somatic mutations in oak found relatively few somatic mutations between distant branches (Schmid‐Siegert et al., 2017; Plomion et al. 2018). Along the same line of thought, branches might differ not only genetically but also epigenetically. Given that epigenetic changes occur at much faster rates (van der Graaf et al., 2015), these differences could be much more pronounced. In addition, the trees in our experiment were grafted on genetically different rootstocks and there is evidence that grafting can alter methylation patterns (Neves et al., 2017; e.g., Wu, Wang, et al., 2013). To determine the effects of somatic mutations or grafting on methylation patterns, future studies should focus on comparisons of methylation patterns of clonal ramets grafted on genetically different rootstocks. Ideally, this should be accompanied by phenotyping and gene expression data.

The relationship between DNA methylation and gene expression is not yet fully understood and vigorously debated (Seymour & Becker, 2017; Zilberman, 2017). Gene body methylation was shown to reduce gene expression in plants as diverse as *Physcomytrella,* maize and poplar (Lang et al., 2018; Li, Song, et al., 2015; Vining et al., 2012). However, the major taxonomic groups of land plants differ substantially in their DNA methyltransferases and also in the characteristics of their GBM (Bewick et al., 2017). Here, our study is in line with other data from conifers (Ausin et al., 2016; Bewick et al., 2017; Takuno et al., 2016) with respect to the distribution of methylation within genes, and also in showing that CHG methylation in gene bodies is quite common compared to many angiosperms at levels similar to CG methylation. This supports the notion that the regulative nature of GBM differs fundamentally among angiosperms and gymnosperms (Niederhuth & Schmitz, 2017). However, thus far there is little data on how methylation and gene expression are linked in gymnosperms. Takuno et al. (2016) found that gene expression in *Pinus taeda* was positively correlated with CG, but not with CHG methylation which indicates a nonrepressive nature for CHG methylation in gene bodies. Also, methylation in regulatory regions located outside of coding regions might critically influence gene expression – and vice versa (Niederhuth & Schmitz, 2017). With our current approach of targeting gene bodies, we obtained around 20 bp up‐ and 17 bp downstream of gene bodies with at least a coverage of 10 ×  (mean upstream: 20.62 bp, SEM: 0.4 for 23,061 genes and mean downstream: 16.55 bp, SEM: 0.34 for 24,212 genes; see Supporting information Table  S3). This would be improved by specifically targeting a priori identified DMRs from WGBS data as suggested above. In addition, a combination of methylation and gene expression data under diverse environmental settings would help in elucidating the interaction of methylation and gene expression.

Seymour and Becker (2017) further stress the fact that most studies used plants grown under common garden conditions, and thus, the extent of DNA methylation variation under natural conditions remains largely unknown. Therefore, future research in plant epigenetics should focus both on broadening the spectrum of plant species in which methylation data are investigated at single‐base resolution, and on investigating methylation patterns under natural environmental conditions. In this context, the application of TBS provides a promising tool for future studies in species with large and complex genomes and permits to integrate a larger number of individuals if natural populations are studied.

## DATA ACCESSIBILITY

The reference.fasta file and.gff files including annotation for introns, exons, and probe regions, as well as information on all methylated positions included in the final analysis, are available at DRYAD under doi:10.5061/dryad.4rn55kq. BAM Files containing the mapped reads are available at European Nucleotide Archive (ENA, http://www.ebi.ac.uk/ena/) under the project PRJEB26494, raw read fastq files are available under the ENA run accessions ERR2591764:ERR2591771.

## AUTHORS CONTRIBUTION

Authors KH, KKU, LO, and SAR provided substantial contributions to the conception and design of the work as well as the acquisition, analysis, or interpretation of data for the work and drafting/revising the work critically for important intellectual content.

Authors MH, SL, RSB, and JZ provided substantial contributions to the acquisition, analysis, and interpretation of data for the work and revised critically for important intellectual content.

All authors gave final approval of the version to be published and agreed to be accountable for all aspects of the work in ensuring that questions related to the accuracy or integrity of any part of the work are appropriately investigated and resolved.
