# Supplementary material for: Detection of somatic epigenetic variation in Norway spruce via targeted bisulfite sequencing
Source: Ecol Evol. 2018 Sep 5;8(19):9672–82. doi: 10.1002/ece3.4374 (PMC6202725; doi:10.1002/ece3.4374)
Supplement: Supplementary file 1 [file ECE3-8-9672-s001.docx]

**Detection of somatic epigenetic variation in Norway spruce via targeted bisulfite sequencing**

Heer, Katrin^1,,62^*; Ullrich, Kristian K.* ^3,4^; Hiss, Manuel^3^; Liepelt, Sascha^1^; Schulze Brüning, Ralf^3^; Zhou, Jiabin^5^; Opgenoorth, Lars^2,©^; Rensing, Stefan A. ^3,7^

^1^ Philipps University Marburg, Faculty of Biology, Conservation Biology, Karl-von-Frisch-Strasse 8, 35043 Marburg, Germany

^2^ Philipps University Marburg, Faculty of Biology, Department of Ecology, Karl-von-Frisch-Strasse 8, 35043 Marburg, Germany

^3^ Philipps University Marburg, Faculty of Biology, Plant Cell Biology, Karl-von-Frisch-Str. 8, 35043 Marburg, Germany

^4^ Max Planck Institute for Evolutionary Biology, Department of Evolutionary Genetics, August Thienemann Str. 2, 24306 Ploen, Germany

^5^ State Key Laboratory of Grassland Agro-Ecosystems, School of Life Sciences, Lanzhou University, 730000 Lanzhou, China

^6^ College of Life Sciences, Shaanxi Normal University, 710119 Xi'an, China

^7^ BIOSS Biological Signaling Studies, University of Freiburg, Germany

**Supporting information**

**Table of contents**

**Figure S1:** Map of the sampling locations of the ortets in the Bavarian Forest National Park and the seed orchard in Übersee.

**Figure S2:** Aerial photo of the sampling sites of the ortets in the Bavarian Forest National Park

**Figure S3:** Aerial photo of the seed orchard in Übersee, Germany were ramets where planted.

**Figure S4:** Climatic differences in mean temperature and precipitation between the sampling sites.

**Figure S5**: Genetic relatedness among ortets based on 1993 SNPs based on a PCoA in GenAlex.

**Figure S6**: Capture efficiency of the targeted bisulfite sequencing approach in Norway spruce.

**Figure S7**: Visualization of GO bias analysis.

**Table S3**: Length of regions (in bp) up- and downstream of annotated gene bodies with at least 10x coverage.


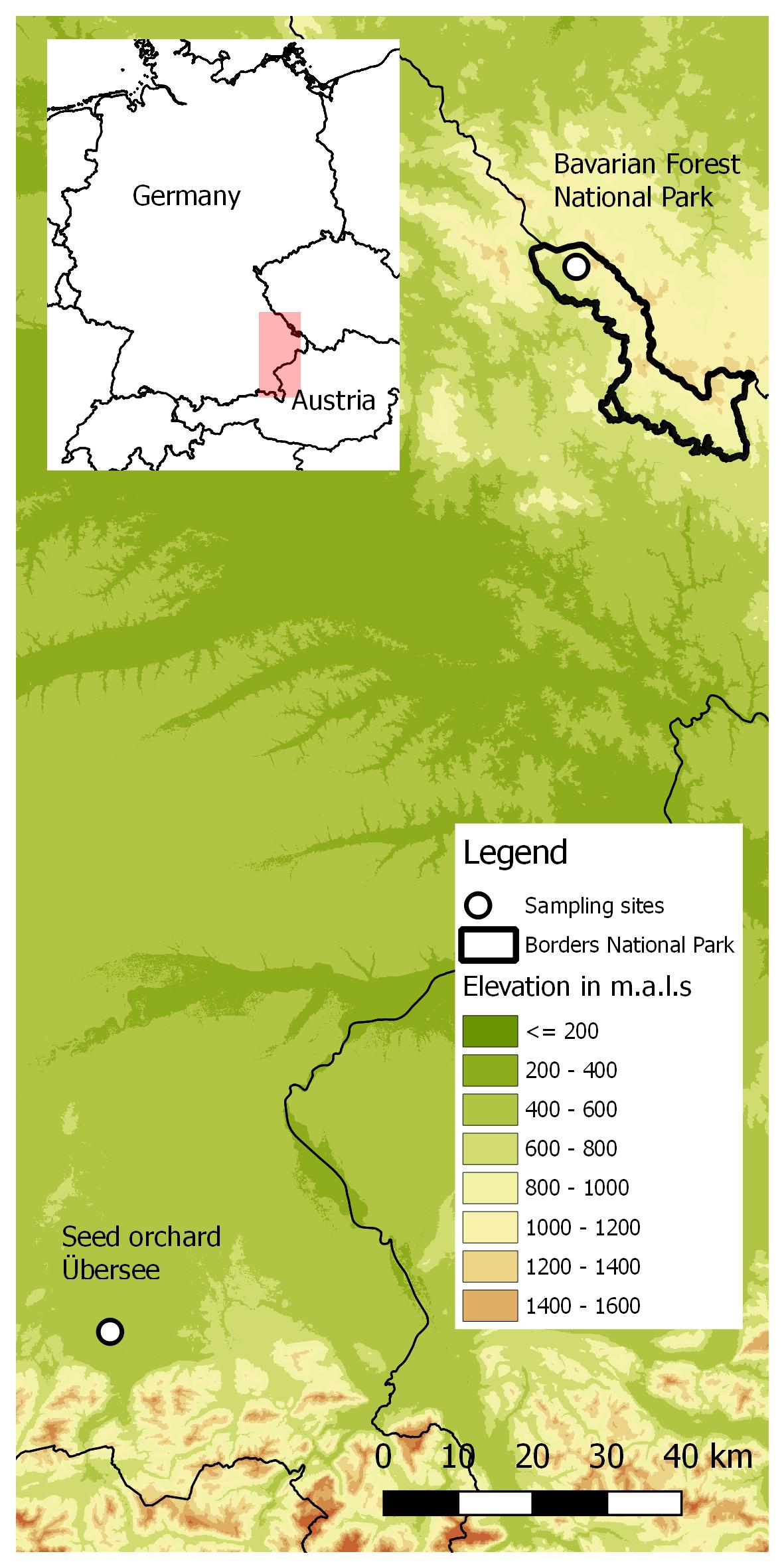


Figure S 1: Map of the sampling locations of the ortets in the Bavarian Forest National Park and the seed orchard in Übersee.


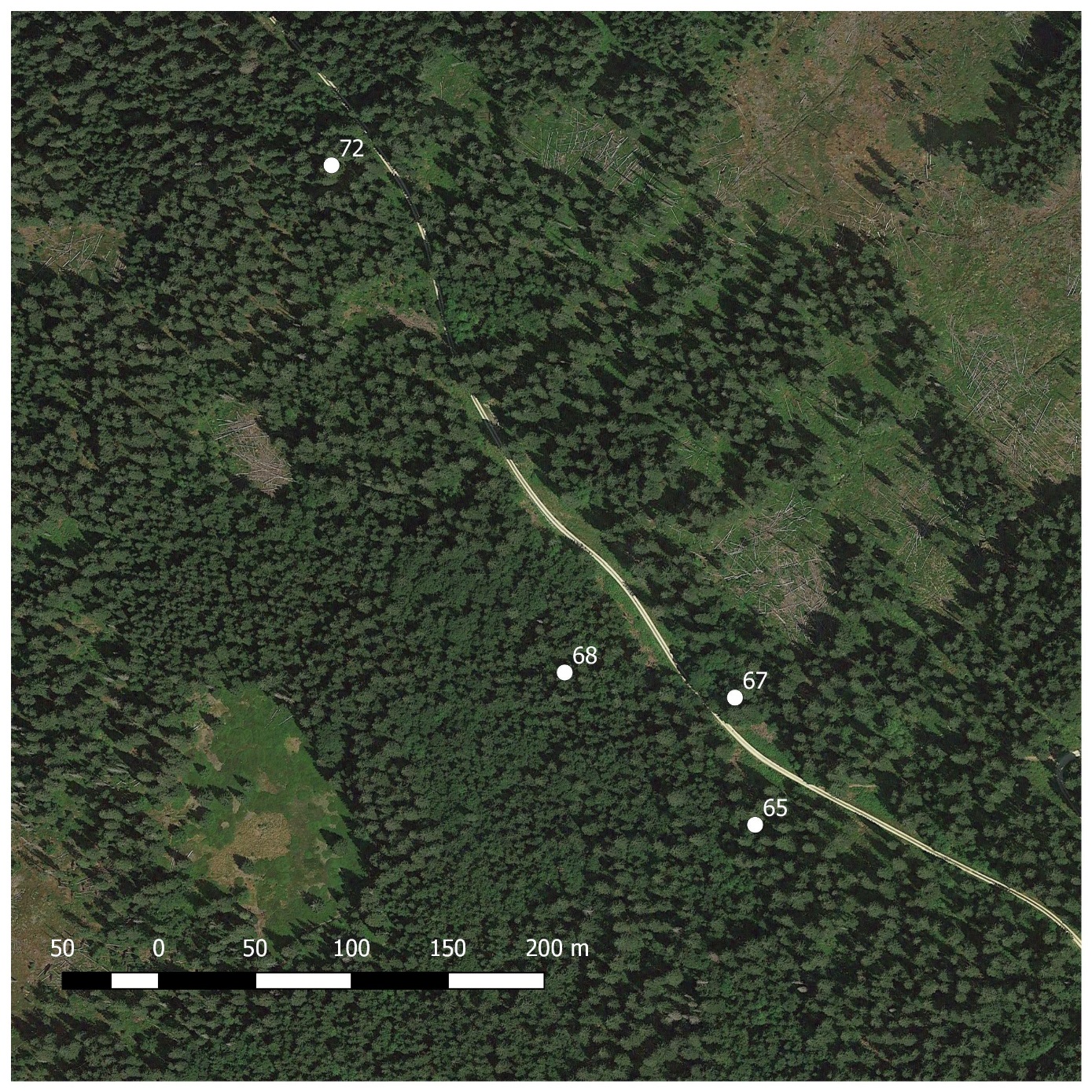


Figure S 2: Aerial photo of the sampling sites of the ortets in the Bavarian Forest National Park (based on Google Satellite)


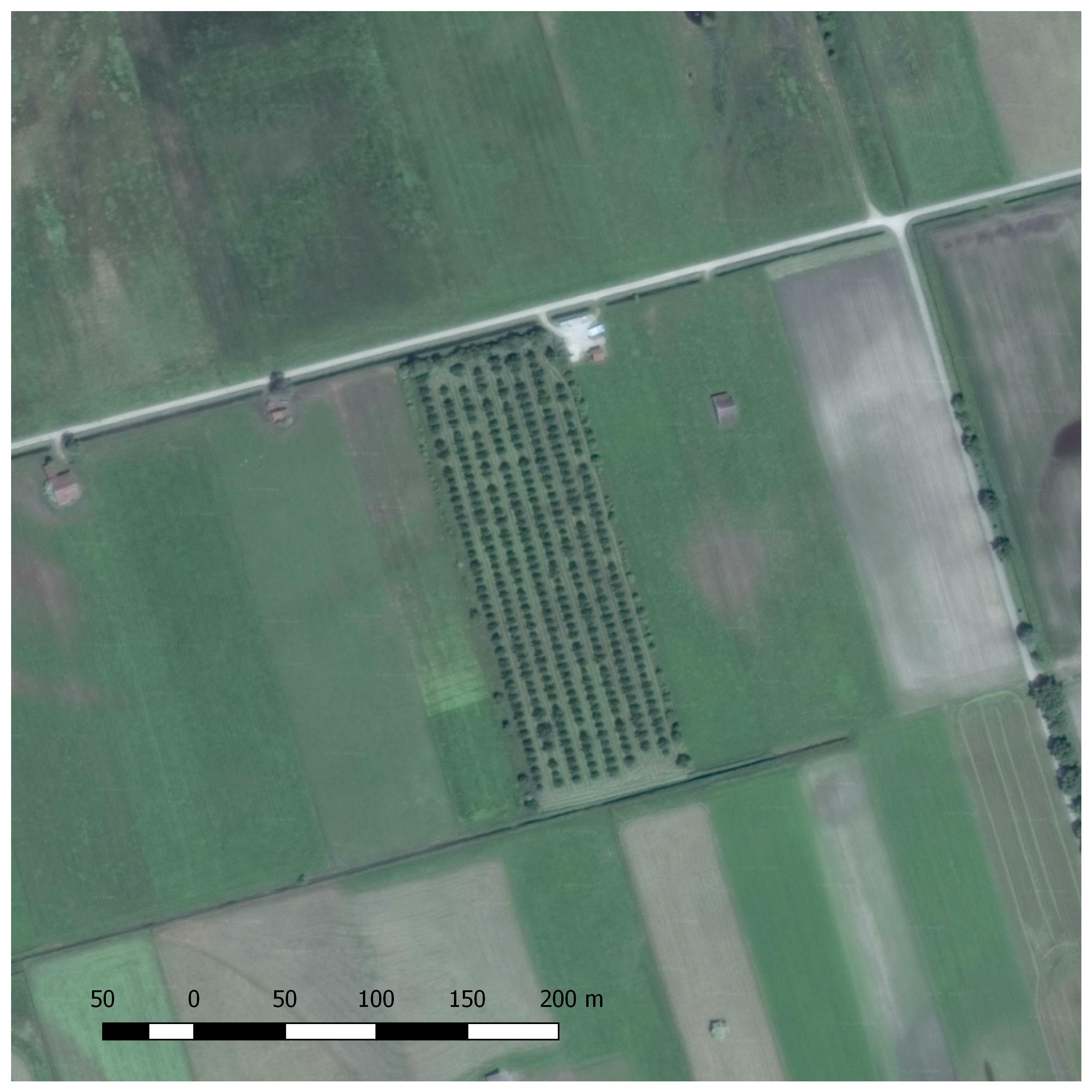


Figure S 3: Aerial photo of the seed orchard in Übersee, Germany were ramets where planted (based on Google satellite).


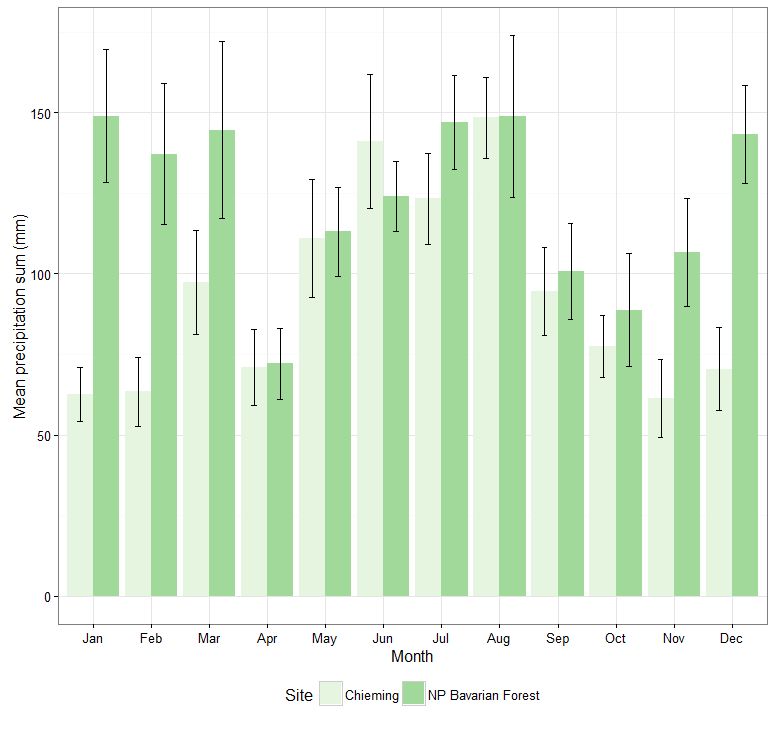

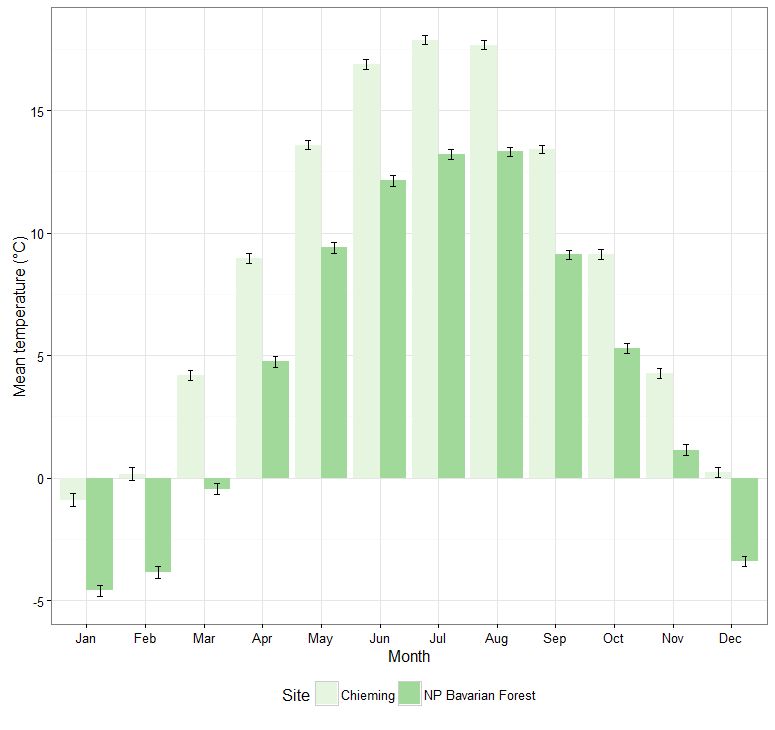


Figure S 4: Climatic differences in mean temperature and precipitation between the sampling sites. Climatic data is obtained from the meteorological station in Chieming which is located close to Übersee, and from a climatic model with a 100 x 100 m resolution from the Bavarian Forest National Park. Mean temperature differs by ~ 4°C. Precipitation differs principally in winter with substantially more snow fall at high elevations.


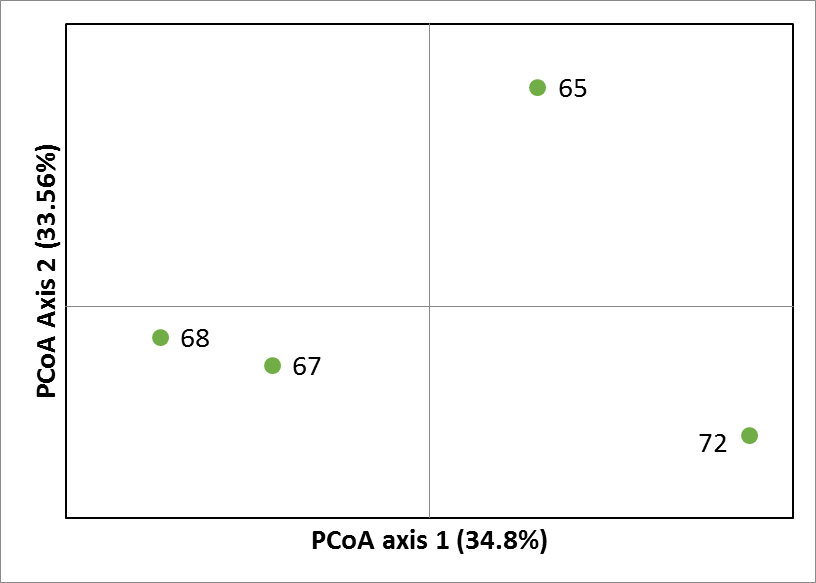


Figure S 5: Genetic relatedness among ortets based on 1993 SNPs based on a PCoA in GenAlex.


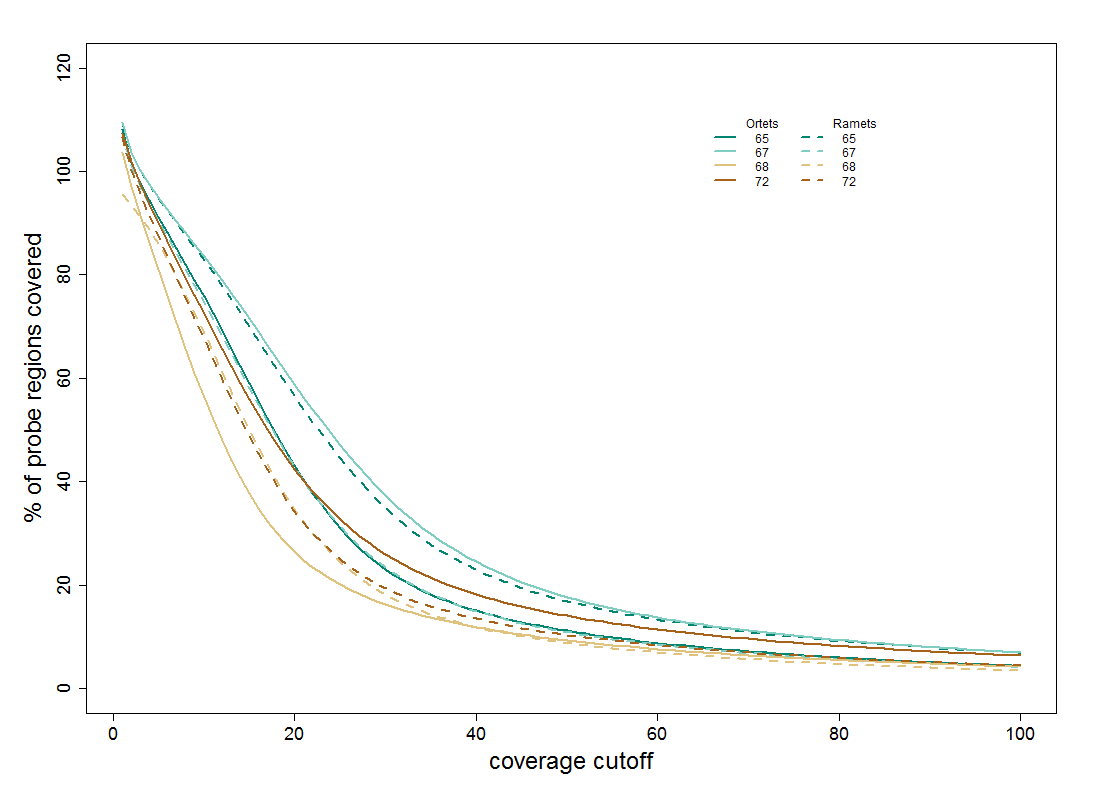


Figure S 6: Capture efficiency of the targeted bisulfite sequencing approach in Norway spruce. The graph depicts the proportion of the genes (TSS to TTS) that are covered with at least the read number indicated on the x-axis. 100% refers to the gene space (TSS to TTS, 89 Mb). As reads were also mapped up and downstream to the premRNA (130 Mb), more than 100% of a given gene can be covered by reads. Colors represent clone IDs. Ortets are represented by quadrats, ramets by triangles.


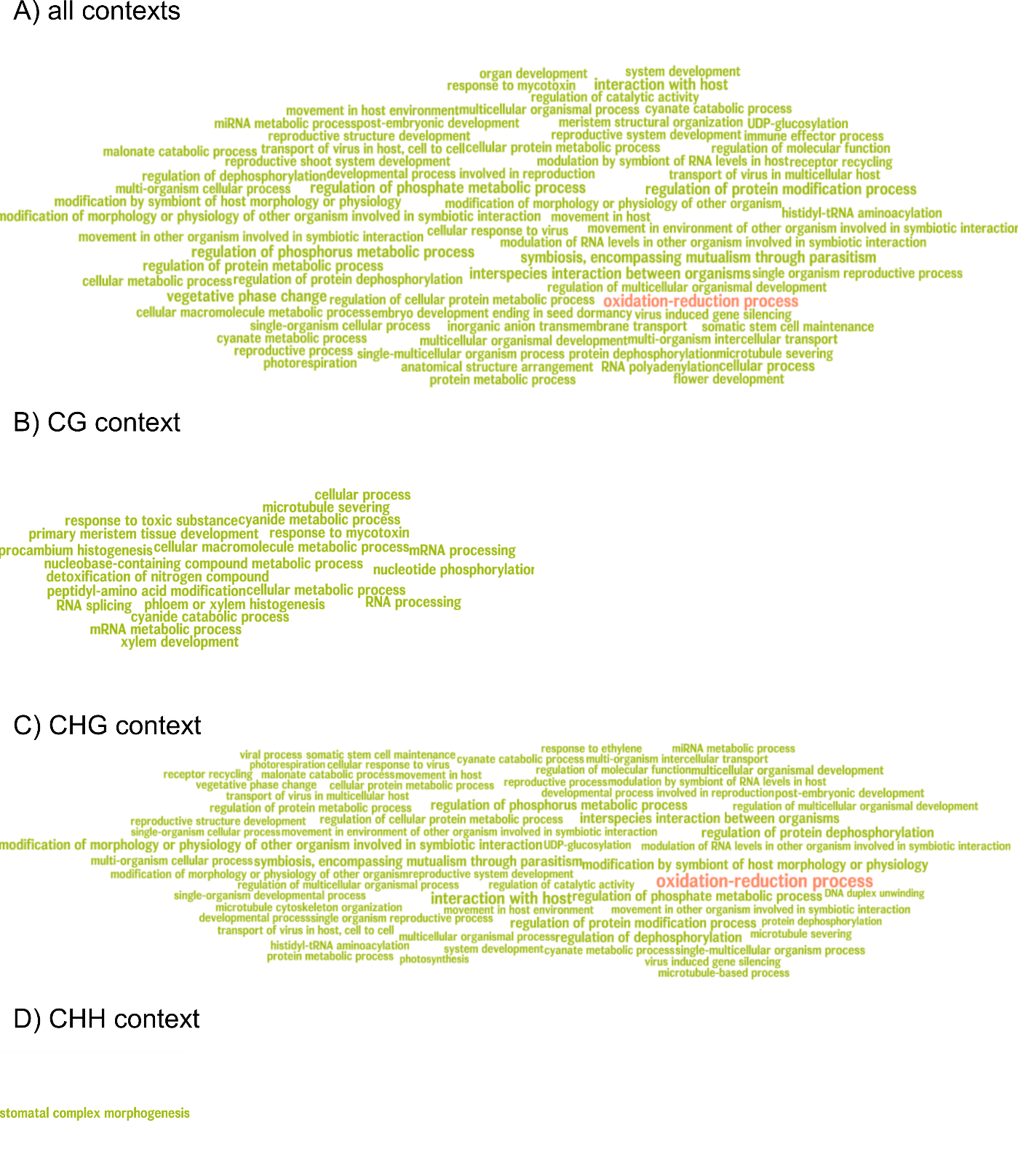


Figure S 7: Visualization of GO bias analysis. Gene Ontology terms of genes harboring DMPs were compared with the full set of high confidence genes. Deviating biological process ontology terms were visualized using word clouds via the www.wordle.net application. Word size corresponds to the fdr-corrected p-value of the term (terms < 0.01 shown). Terms enriched in the DMP set are shown in green, those depleted are shown in red. Figures depict deviating GO terms for A) all contexts combined, and for the B) CG, C) CHG and D) CHH context separately.

Table S3: Length of regions (in bp) up- and downstream of annotated gene bodies with at least 10x coverage. First position smaller than 10x coverage was used to define region length. The mean is calculated for N (number of genes with coverage outside annotated gene body) regions per library. SEM: Standard deviation of the mean.

| **10x coverage** | **ortet65** | **ortet67** | **ortet68** | **ortet72** | **ramet65** | **ramet67** | **ramet68** | **ramet72** | **AVERAGE** |
| --- | --- | --- | --- | --- | --- | --- | --- | --- | --- |
| Mean – upstream | 19.87 | 28.10 | 13.19 | 23.12 | 26.56 | 19.87 | 17.59 | 16.64 | 20.62 |
| SEM- upstream | 0.39 | 0.46 | 0.33 | 0.46 | 0.44 | 0.38 | 0.39 | 0.37 | 0.40 |
| N – upstream | 23066 | 23046 | 23076 | 23056 | 23049 | 23061 | 23065 | 23068 | 23061 |
| Mean – downstream | 16.01 | 21.61 | 10.81 | 18.23 | 21.38 | 16.55 | 14.26 | 13.52 | 16.55 |
| SEM – downstream | 0.32 | 0.39 | 0.28 | 0.37 | 0.38 | 0.33 | 0.32 | 0.31 | 0.34 |
| N – downstream | 24214 | 24203 | 24216 | 24210 | 24205 | 24211 | 24218 | 24217 | 24212 |
