# Supplementary material for: Detection of somatic epigenetic variation in Norway spruce via targeted bisulfite sequencing
Source: Ecol Evol. 2018 Sep 5;8(19):9672–82. doi: 10.1002/ece3.4374 (PMC6202725; doi:10.1002/ece3.4374)
Supplement: Supplementary file 4 [file ECE3-8-9672-s004.docx]

Supporting Information

**Table S1**: Mean coverage per probe and annotated gene model for CG, CHG and CHH methylation context, after filtering for coverage of at least 8 prior methylation calling. See methods section for details on filtering, methylation calling and feature annotation.

**Table S2**: Information on the DMPs identified with MethylKit, and on the subset for which the methylation differed consistently by at least 10% between ortet and ramet. For each context, we provide a table that contains the following information for each DMP and library: coverage, C count, T count and percent methylation. In addition, we list the p and q values from MethylKit, the percentage difference between each ortet-ramet pair and indicate whether the threshold of 10% was passed for all ortet-ramet pairs.
